# Propagation and Selectivity of Axonal Loss in Leber Hereditary Optic Neuropathy

**DOI:** 10.1038/s41598-019-43180-z

**Published:** 2019-04-30

**Authors:** Razek Georges Coussa, Pooya Merat, Leonard A. Levin

**Affiliations:** 1 Department of Ophthalmology and Visual Sciences, McGill University, Montreal, Canada; 2 Department of Electrical and Computer Engineering, McGill University, Montreal, Canada; 3 Department of Neurology & Neurosurgery, McGill University, Montreal, Canada; 4 Department of Ophthalmology and Visual Sciences, University of Wisconsin, Madison, WI USA

**Keywords:** Hereditary eye disease, Optic nerve diseases

## Abstract

Leber hereditary optic neuropathy (LHON) is a syndrome of subacute loss of central vision associated with mutations in mitochondrial DNA coding for components of complex I. LHON preferentially involves small axons in the temporal optic nerve, but the reason is unclear. We performed a Monte Carlo simulation of the spread of injury in LHON axons to better understand the predilection for small axons. Optic nerve slices were modeled as grids containing axons with sizes from reported regional distributions. The propagation of injury from a localized concentration of superoxide was simulated as the spread via passive diffusion from one axon to adjacent axons, with basal production and scavenging rate proportional to axonal area and volume, respectively. Axonal degeneration occurred when intra-axonal concentrations reached a toxic threshold. Simulations demonstrated that almost all small and medium axons degenerated by the time steady-state was reached, but about 50% of large axons were preserved. The location of initial injury affected time to steady state, with nasal injuries reaching steady state faster than temporal injuries. The pattern of axonal degeneration in the simulations mirrored both visual fields and optic nerve histology from patients with LHON. These results provide insight into the nature of axonal loss in LHON.

## Introduction

Leber hereditary optic neuropathy (LHON) is a mitochondrial optic neuropathy which predominantly affects men in their late teens or early twenties, but can affect both sexes at any age^1^. The prevalence in England and Finland is 1 in 30–50,000 individuals^2–4^. LHON is characterized by three clinical phases: presymptomatic, acute, and atrophic. In the presymptomatic phase, patients do not have frank visual loss but may have reduced contrast sensitivity and red-green color impairment^5^. Fundus examination may reveal peripapillary telangiectatic vessels and/or retinal nerve fiber layer (RNFL) pseudo-edema, particularly in the papillomacular bundle^6,7^. The electroretinogram can also be reduced^5^.

The acute phase of the disease is typically characterized by unilateral painless central visual acuity loss to counting fingers or worse. Visual field testing generally reveals central or cecocentral scotomas, likely due to the involvement of the papillomacular bundle^8^. In 75% of cases, the contralateral eye becomes similarly affected 6–8 weeks after the initial event^6^. The central or cecocentral visual field defects can progressively enlarge over time^9^. Eventually there is optic atrophy, and visual loss is usually permanent.

More than 95% of LHON patients display point mutations in mitochondrial DNA (mtDNA) at one of three positions: 11778 (which has the worst long-term visual prognosis), 14484 (which is associated with the best prognosis), and 3460 (which is intermediate with respect to visual prognosis). All 3 mutations code for components of the mitochondrial complex I electron transport chain, which oxidizes NADH by serving as an NADH:ubiquinone oxidoreductase^8^. Other mutations in genes coding for complex I are also seen, albeit at lower frequencies.

The triggering factors responsible for progression from the presymptomatic to the acute phase are not well understood, and have been hypothesized to include tobacco smoking and alcohol consumption^10^, vitamin B_12_ deficiency^11^, head trauma^12^, and increased intraocular pressure^13^.

LHON displays specific clinical features that are shared by a limited number of other diseases, namely cecocentral scotomas (an uncommon pattern of visual field defect), dyschromatopsia, and symmetric and severe bilateral loss of visual acuity. These features are also seen in vitamin B_12_ deficiency, some toxic optic neuropathies, and dominant optic atrophy. The similar clinical pattern in these diseases is likely due to the selective involvement of small diameter axons within the optic nerve^8^.

Consistent with this hypothesis, Pan *et al*. described an orderly pattern of axonal loss in LHON, starting inferotemporally and then progressing centrally, based on post-mortem analysis of optic nerve cross sections^14^. In LHON, the superonasal axons are affected late in the natural history of the disease^14^. These findings parallel the size distribution of axons in the human optic nerve, with inferotemporal axons having the smallest radii and superonasal axons having a heterogeneous distribution skewed to larger radii^14^.

The relation between the selectivity of LHON for small axons and its pathophysiology is unclear. Superoxide anion, an oxygen free radical generated in multiple cellular processes, may be a critical component in LHON pathophysiology. Superoxide is produced at high levels when LHON mtDNA mutations are expressed in cybrids^15^, and has an important role in the signaling of axonal injury. It is reported to increase in the soma when the axons are injured, consequently leading to apoptosis^16,17^. In LHON, the dysfunctional complex I loses its oxidizing ability and cannot transfer electrons to ubiquinone from NADH. Superoxide is formed when these electrons reacts with molecular oxygen^8^. The increase in superoxide production could falsely signal axonal injury and ultimately lead to death of retinal ganglion cells in LHON^8,18^.

We hypothesized that the preferential small axon involvement in LHON reflects the combination of (1) an increased production of superoxide in LHON axons; and (2) size-dependent rates of superoxide generation and scavenging. To test this hypothesis, we simulated the spread of an initial area of increased superoxide (the “injury”) within a modeled optic nerve using a Monte Carlo approach.

## Results

### Optic nerve axon distribution

Modeling of optic nerve pathophysiology required that the number and size distribution of axons be realistic, specifically that the number of small, medium and large axons needed to match that of the human optic nerve. For computational reasons, most simulations were performed with an optic nerve that was 10% of the radius (1% of the area) of the human optic nerve. Given that the human optic nerve contains approximately 1,200,000 axons^19^, most simulations were performed with approximately 12,000 axons. The distributions of small, medium and large axons (each one tertile) across the nerve at the initial state matched that extrapolated from histopathological data derived from normal nerves reported by Pan *et al*.^14^ (their Fig. 3). By fitting the data with a three-component Gaussian function, we were able to model a scaled version with 12104 ± 169 axons, based on generating 30 sample optic nerves. The final parameters of the Gaussian function are listed in Supplementary Table 1. The overall adjusted R^2^ of the Pearson correlation coefficient between the three-component Gaussian function and the actual axonal size data from Pan *et al*. was 99.8%, illustrating the accuracy of the model.

### Output from simulation

**Figure 1:**
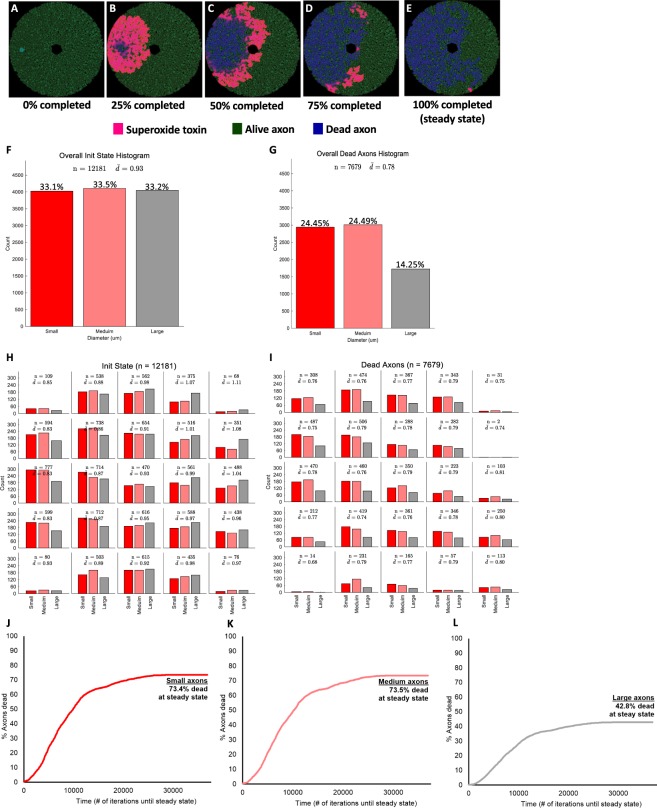
Example of simulation of LHON optic nerve degeneration. (**A**) Schematic of the optic nerve at the initial state (t = 0). (**B**) Simulation at 25% completion. (**C**) Simulation at 50% completion. (**D**) Simulation at 75% completion. (**E**) Simulation at 100% completion (at steady state). **(F)** Initial axon size distribution. (**G**) Steady-state axonal size distribution. (**H**) Initial axonal size distribution by location. (**I**) Steady-state axonal size distribution by location. (**J**) Proportion of small degenerated axons over time. (**K**) Proportion of medium degenerated axons over time. (**L**) Proportion of large degenerated axons over time.

Figure 1 illustrates an example of a simulation of a 10% (radius) scaled version of the optic nerve, with 12181 axons at the initial state. Figure 1A–E and Supplementary Video 1 demonstrate graphically the diffusion of superoxide and the propagation of damage, from the initial to the steady state. The diffusion of superoxide is depicted in pink, while live and degenerated axons are depicted in green and blue, respectively. In this example, the initial superoxide injury was placed in the temporal optic nerve (Fig. 1A) and then progressively spread superotemporally and inferotemporally (Fig. 1B–D). At steady state (Fig. 1E), most temporal axons had degenerated (blue), while a sizable portion of the nasal ones were still alive (green). Figure 1F to I represent the axonal size distribution at the initial (Fig. 1F,H) and final states (Fig. 1G,I). By definition, the initial proportions of small, medium and large axons were equal (Fig. 1F) with a preferential larger number of smaller axons located temporally (red bars on Fig. 1H). There was a larger proportion of large axons (grey bars on Fig. 1H) in the nasal half of the modeled optic nerve.

Once steady state was reached, 24.4%, 24.5% and 14.2% of small, medium and large axons had degenerated, respectively (Fig. 1G), with preferential loss of smaller axons located temporally (Fig. 1I). Across all simulations, when the initial injury was located temporally, there were more degenerated axons in the temporal half of the optic nerve compared to the nasal one (Fig. 1E,I). Figure 1J to L present the proportion of degenerated axons as the simulation progresses, represented by the total number of iterations until steady state. At steady state, 62.6 ± 3.0% of all axons degenerated, representing 73.2 ± 3.5%, 72.3 ± 3.2% and 42.4 ± 2.6% of small, medium and large axons, respectively (Fig. 1J–L).

These simulations demonstrated that smaller axons are more likely to die than larger axons, and that initial injuries located in the temporal part of the optic nerve (where there are more small axons) are likely to spread and involve much of the temporal optic nerve. These findings match those observed clinically and pathologically^14^.

### Sensitivity analysis of varying simulation parameters

To ensure that the model was pathophysiologically realistic, the following parameters were systematically varied: location of the initial injury, the intra-axonal and extra-axonal superoxide scavenging constants, and the superoxide production constant. In addition, the simulation was repeated for different optic nerves.

#### Injury location

**Figure 2:**
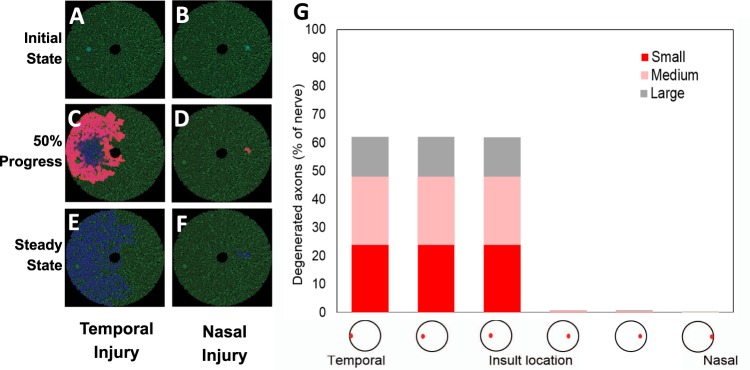
Sensitivity analysis on superoxide injury location varying along the temporal-nasal axis. (**A**) Schematic of the optic nerve with a temporal injury at the initial state (time = 0). (**B**) Simulation at 50% completion. (**C**) Simulation at 100% completion (at steady state). (**D**) Schematic of the optic nerve with a nasal injury at the initial state (time = 0). (**E**) Simulation at 50% completion. (**F**) Simulation at 100% completion (at steady state). (**G**) Steady-state axonal size distribution.

**Figure 3:**
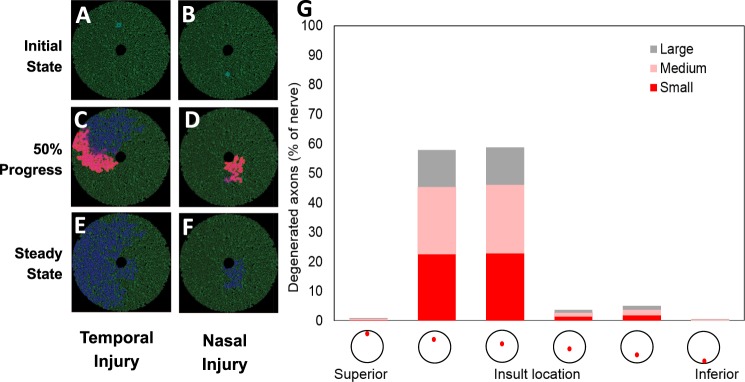
Sensitivity analysis on superoxide injury location varying along the superior-inferior axis. (**A**) Schematic of the optic nerve with a superior injury at the initial state (time = 0). (**B**) Simulation at 50% completion. (**C**) Simulation at 100% completion (at steady state). (**D**) Schematic of the optic nerve with an inferior injury at the initial state (time = 0). (**E**) Simulation at 50% completion. (**F**) Simulation at 100% completion (at steady state). (**G**) Steady-state axonal size distribution.

Sensitivity analyses on various injury locations were performed to characterize the superoxide diffusion pattern as a function of the geographical axonal size distribution. For the purpose of this sensitivity analysis, we used the same optic nerve model and changed the injury location along the temporal-nasal axis (Fig. 2, Supplementary Table 2) and the superior-inferior axis (Fig. 3, Supplementary Table 3). Figure 2A,C,E and Supplementary Video 1 illustrate the schematic evolution of the superoxide diffusion rate when the initial injury was located in the temporal optic nerve, and Fig. 2B,D,F and Supplementary Video 2 illustrate it when the initial injury was located in the nasal optic nerve. When the injury was nasal, there was far less propagation of superoxide, and hence degenerated axons, than when it was temporal (Fig. 2A,C,E vs B,D and F). This finding is supported by the proportion of degenerated axons at steady state (Fig. 2G, Supplementary Table 2). When the initial injury was varied from far temporal to mid-temporal to temporal, the proportions of small, medium and large degenerated axons were: 24/24/14%, 24/24/14%, and 24/24/14%, respectively (first three columns in Fig. 2G, Supplementary Table 2). In contrast, when the initial injury was varied from nasal to mid-nasal and then to far-nasal, the proportions of small, medium and large degenerated axons were much lower at (0.35/0.37/0.03%, 0.24/0.32/0.23%, and 0.16/0.06/0.27%, respectively; last three columns in Fig. 2G, Supplementary Table 2).

Similarly, the propagation of superoxide displayed a similar dichotomous pattern when the initial injury was varied along the superior-inferior axis (Fig. 3A,C,E vs B,D and F). When the injury was located superiorly or mid-superiorly, there was a greater propagation of superoxide and consequently more degenerating axons. In particular, 23/23/13% of the axons in the superior or mid-superior locations, respectively, had degenerated (Fig. 3G, Supplementary Table 3).

#### Superoxide-related constants

The rate of superoxide scavenging (detoxification) plays an important role in influencing the diffusion of superoxide, and consequently should influence the steady-state proportion of degenerated axons. Indeed, when the rate of superoxide scavenging within axons (intra-axonal scavenging) was varied from 0.0025 to 0.05 µmol/iteration, the proportion of degenerated axons decreased from 99% to 0.1% (Supplementary Fig. 2 and Supplementary Table 4). The extra-axonal scavenging rate also affected axonal degeneration. When the extra-axonal scavenging rate was varied from 10^−6^ to 0.05 µmol/iteration, the proportion of degenerated axons decreased from 81% to 0.4% (Supplementary Fig. 3 and Supplementary Table 5).

The amount of superoxide release associated with a degenerating axon was also found to influence the propagation of injury. This was confirmed in a sensitivity analysis performed on the superoxide release constant (µmol per degenerating axon), demonstrating an increase in the proportion of degenerated axons (from 0.2% to 98.3%) when the constant was increased from 1,000 to 30,000 (Supplementary Fig. 4 and Supplementary Table 6).

#### Simulation repetition

**Figure 4:**
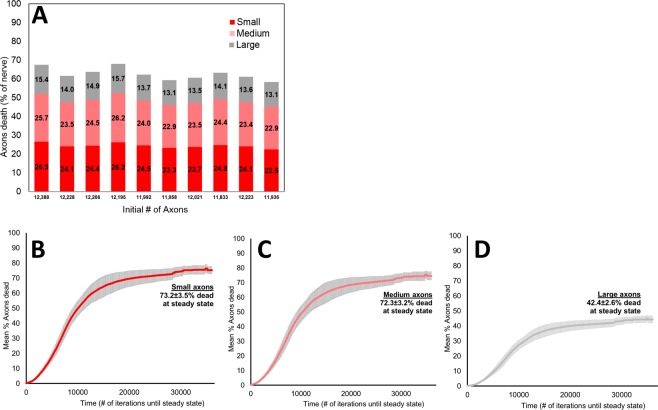
Degenerated axonal size distribution for 10 different 10% scaled optic nerves with constant simulation parameters and middle temporal injury. (**A**) Steady-state axonal size distribution for each simulation run. (**B**) Mean proportion of small degenerated axons over time. (**C**) Mean proportion of medium degenerated axons over time. (**D**) Mean proportion of large degenerated axons over time.

In order to assess the robustness of the model, we studied the variability of the results when it was repeated with different simulated optic nerves and different initial injuries in the same general location. When the simulation was repeated 10 times, with a mean initial number of axons of 12104 ± 169 (Supplementary Table 7), the mean steady-state proportions of degenerated small, medium and large axons were 24.4 ± 1.2%, 24.1 ± 1.1%, and 14.1 ± 0.9%, respectively, i.e. there was relative sparing of larger axons (Fig. 4A and Supplementary Table 7). At steady state, 62.6 ± 3.0% of all axons degenerated. Correspondingly, 73.2 ± 3.5%, 72.3 ± 3.2%, and 42.4 ± 2.6% of small, medium and large axons degenerated at the end of the simulation (Fig. 4B–D). A one-way ANOVA comparing the proportions of degenerated axons within each size category among the 10 runs was not significant (p = 0.99, Supplementary Table 7).

#### Higher optic nerve scaling

20%-Scale Model: Most experiments simulated the optic nerve using a 10% diameter model (1% of the number of axons) because of computational complexity, despite the use of a massively parallel GPU-based platform. To assess whether this scaling produced spurious results, we repeated some studies with a 20%-scale version. Superoxide diffusion and propagation of axonal degeneration were studied using ten independent simulation runs, all with an initial temporal injury. The schematic representation of the superoxide diffusion is illustrated in Supplementary Fig. 5A–C, where the propagation injury stopped nasally at steady state (Supplementary Fig. 5C). The mean initial number of axons was 48560 ± 250 (Supplementary Table 8), and 59.0 ± 3.2% of all axons had degenerated at steady state. Supplementary Fig. 5D depicts the specific proportions of degenerated axons by size at steady state for each run. Similar to the 10%-scale version, the mean steady state proportions of degenerated small, medium and large axons were 23.1 ± 1.3%, 22.9 ± 1.3, and 13.1 ± 0.8%, respectively (Supplementary Fig. 5D and Supplementary Table 8). The proportions of degenerated axons by size over time followed a similar pattern to those in the 10%-scale version, with smaller axons being affected to a greater extent (Supplementary Fig. 5E,F). Specifically, 69.3 ± 3.8%, 68.6 ± 3.7%, and 39.1 ± 2.2% of small, medium and large axons had degenerated by the end of simulation (Supplementary Fig. 5E,F). A one-way ANOVA comparing the proportions of degenerated axons within each size category among the ten runs was not significant (p = 0.99, Supplementary Table 8). A one-way ANOVA comparing the proportions of degenerated axon within each size category among the ten runs for the 10% and 20% scaled versions was also not significant (p = 0.35).

50%-Scale Model: When the model was scaled to 50% of the actual optic nerve size, the simulated number of axons was 304178 axons. Due to computational limitations, we ran a single version of the 50% scale with a temporal injury (Supplementary Fig. 6).

At steady state, the proportions of degenerated small, medium and large axons with the 50% model were 21.7%, 21.3%, and 11.9%, respectively. These values are similar to those seen with the 10% model (small, medium and large: 24.4 ± 1.2%, 24.1 ± 1.1%, and 14.1 ± 0.9%, respectively) and 20% model (small, medium and large axons: 23.1 ± 1.3%, 22.9 ± 1.3%, and 13.1 ± 0.8%, respectively). These results suggest that the simulation and its scalability were robust.

### *In vivo* model validation

**Figure 5:**
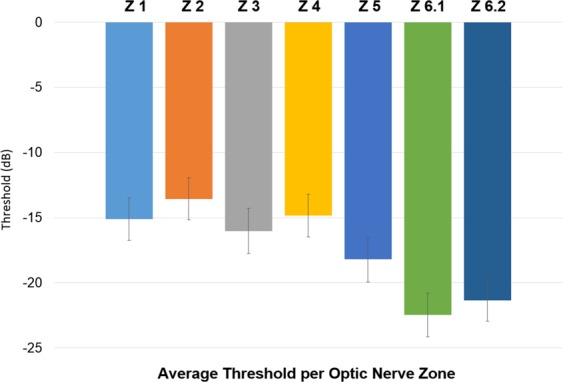
Average mean deviation threshold distribution per Garway-Heath optic nerve zones. Data obtained from 30-2 visual fields from 43 LHON patients.

In order to validate the clinical relevance of our model and results, we compared visual field data from de-identified patients with LHON to the anatomical distribution of simulated degenerated optic nerve axons at steady state, based on 20 different initial injury locations. The optic nerve was divided into 7 zones, based on the Garway-Heath mapping of the correlation between optic nerve head and visual field^20^. Visual fields from 43 LHON patients (35 SITA-FAST 30-2 and 8 FASTPAC 30-2) were used. The mean age of the patients was 37 ± 16 years. The means of the visual field mean deviations were −15.1 ± 1.6, −13.6 ± 1.6, −16.0 ± 1.7, −14.8 ± 1.6, −18.2 ± 1.7, 22.5 ± 1.7, and −21.3 ± 1.6 for zones 1, 2, 3, 4, 5, 6.1 and 6.2, respectively (Fig. 5), with the thresholds increasing in a radial pattern from zone 1 on the nasal aspect of the optic nerve to zone 6 on the temporal side.

**Figure 6:**
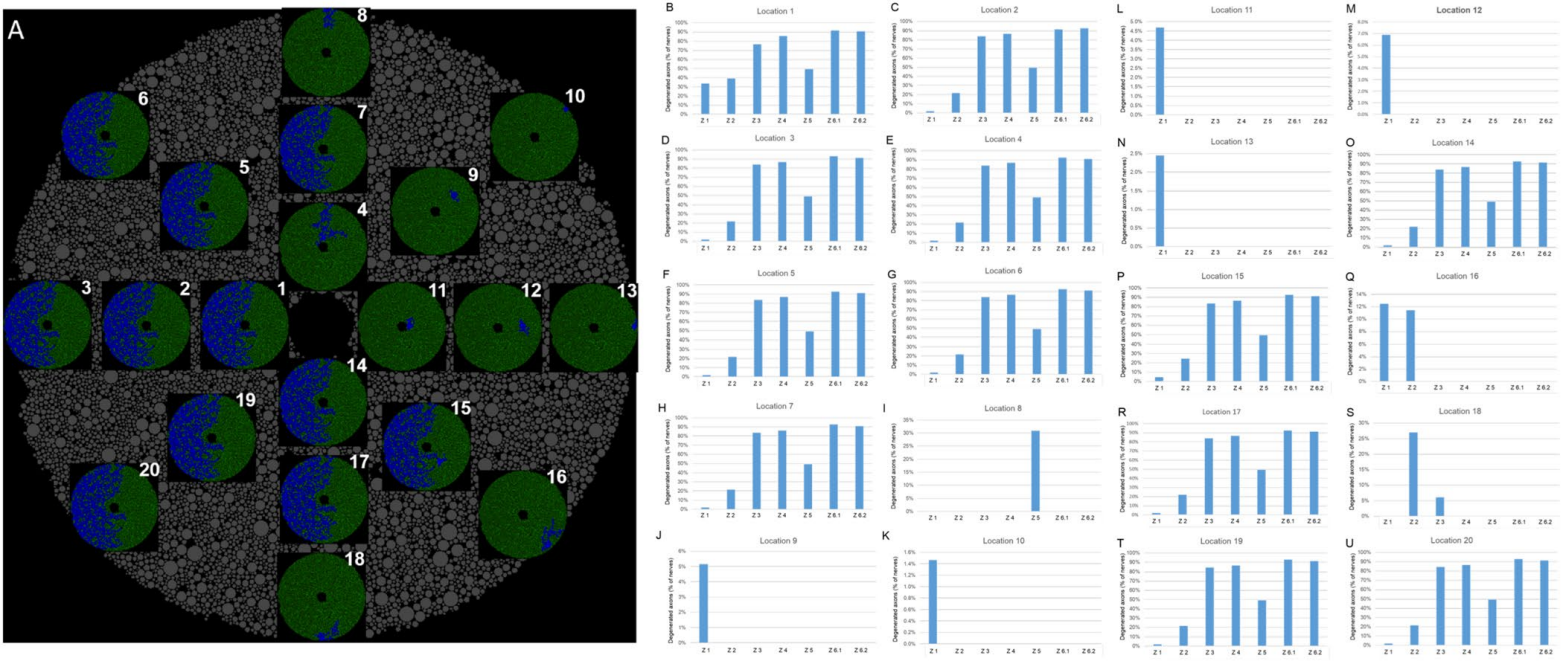
10%-scale optic nerve model simulation validation. (**A**) Schematic of the 20 simulated initial injury locations, their respective steady state superoxide propagation profile and the final proportion of degenerated axons (top right of each optic nerve simulation). (**B**) Proportion of steady state degenerated axons by Garway-Heath optic nerve zones for initial injury location 1. (**C**) Proportion of steady state degenerated axons by Garway-Heath optic nerve zones for initial injury location 2. (**D**) Proportion of steady state degenerated axons by Garway-Heath optic nerve zones for initial injury location 3. (**E**) Proportion of steady state degenerated axons by Garway-Heath optic nerve zones for initial injury location 4. (**F**) Proportion of steady state degenerated axons by Garway-Heath optic nerve zones for initial injury location 5. (**G**) Proportion of steady state degenerated axons by Garway-Heath optic nerve zones for initial injury location 6. (**H**) Proportion of steady state degenerated axons by Garway-Heath optic nerve zones for initial injury location 7. (**I**) Proportion of steady state degenerated axons by Garway-Heath optic nerve zones for initial injury location 8. (**J**) Proportion of steady state degenerated axons by Garway-Heath optic nerve zones for initial injury location 9. (**K**) Proportion of steady state degenerated axons by Garway-Heath optic nerve zones for initial injury location 10. (**L**) Proportion of steady state degenerated axons by Garway-Heath optic nerve zones for initial injury location 11. (**M**) Proportion of steady state degenerated axons by Garway-Heath optic nerve zones for initial injury location 12. (**N**) Proportion of steady state degenerated axons by Garway-Heath optic nerve zones for initial injury location 13. (**O**) Proportion of steady state degenerated axons by Garway-Heath optic nerve zones for initial injury location 14. (**P**) Proportion of steady state degenerated axons by Garway-Heath optic nerve zones for initial injury location 15. (**Q**) Proportion of steady state degenerated axons by Garway-Heath optic nerve zones for initial injury location 16. (**R**) Proportion of steady state degenerated axons by Garway-Heath optic nerve zones for initial injury location 17. (**S**) Proportion of steady state degenerated axons by Garway-Heath optic nerve zones for initial injury location 18. (**T**) Proportion of steady state degenerated axons by Garway-Heath optic nerve zones for initial injury location 19. (**U**) Proportion of steady state degenerated axons by Garway-Heath optic nerve zones for initial injury location 20.

Figure 6A illustrates the 20 simulated initial injury locations and the corresponding profile of superoxide propagation at steady state. Supplementary Table 9 lists the steady state proportions of degenerated axons for each simulated run. Similar to what was found previously, when the initial injury was more nasal, the number of degenerated axons at steady state was lower. Figure 6B to U depict the steady state proportions of degenerated axons grouped by Garway-Heath optic nerve’ zones. For initial injuries located temporally (zones 3, 4 and 6), the steady state proportion of degenerated axons was approximately 60%, regardless of the location of the initial injury. This is consistent with a significant propagation of superoxide in initial temporal injuries. Furthermore, the distribution of axons by zones for these same locations (location 1, 2, 3, 4, 7, 14, 17, 19 and 20, Fig. 6A–D,F–H,O,R,T and U) was consistent and reproducible. Specifically, 5 ± 0.1%, 25 ± 0.1%, 83 ± 0.0%, 86 ± 0.0%, 49 ± 0.0%, 92 ± 0.0% and 91 ± 0.0% of axons in zones 1, 2, 3, 4, 5, 6.1 and 6.2, respectively, had degenerated at steady state when the initial injury was located within the temporal half of the simulated optic nerve. When the initial injury was instead located within the nasal half of the optic nerve (zones 1, 2 and 5), the simulated propagation of superoxide was substantially slower, and the axonal degeneration remained localized within the same zone as the initial injury (Fig. 6J–N,Q).

## Discussion

We performed a Monte Carlo simulation of the spread of superoxide, a putative axonal toxin, in a 2-dimensional model of the human optic nerve. Axons exposed to a threshold concentration of superoxide were marked as degenerating. For an initial temporal injury, the pattern of propagation of superoxide and degenerating axons was consistent with small and medium-sized axons being affected first and to a greater extent than large axons. At steady state, approximately 73% of small and medium-sized axons degenerated, compared to 42% of large ones. These findings were consistent when repeated with similar modeled optic nerves or nerves with more axons (Supplementary Tables 7 and 8). This supports the robustness, repeatability and scalability of the optic nerve and superoxide diffusion models used in this study.

The modeled simulated axonal size distribution was based on actual optic nerve measurements from a published study^14^, with smaller and larger axons preferentially located temporally and nasally. This asymmetric distribution proved to be critical for the nature of superoxide diffusion and subsequent axonal degeneration. The importance of the axonal size distribution was highlighted by the sensitivity analyses carried on the initial superoxide injury location. For initial injuries located temporally and/or superiorly, where small and medium axons are preferentially located, about 60% of all axons degenerated compared to 2% (with only 0.5% degeneration of large axons, i.e. relative sensitivity of smaller axons) for initial injuries located nasally or inferiorly. Compared to large axons, smaller ones were 1.7 times more likely to die when the initial injury was located in areas containing more small axons.

Sensitivity analyses on the scavenging constants supported the representative nature of the model. Larger scavenging constants resulted in substantially greater axonal viability, i.e. when the intra-axonal scavenging constant increased 10-fold from 0.005 to 0.05 µmol/iteration, the overall proportion of degenerated axons decreased by a factor of 874 (Supplementary Table 4). Similar results were found for the extra-axonal scavenging constant, where a 10-fold increase resulted in a 166-fold decrease in the proportion of degenerated axons (Supplementary Table 5). The relative effect of the intra-axonal scavenging constant on axonal viability was about 5 times larger than that of the extra-axonal constant (874 vs. 166), presumably reflecting the critical role of intra-axonal superoxide concentrations on axonal degeneration.

As expected, the sensitivity analysis of the amount of superoxide released when an axon degenerates demonstrated that there was a level beyond which virtually all axons degenerated. This reflects the limited amount of superoxide scavenging in a biological system. The scalability of the model was excellent, with the proportion of degenerated axons remaining essentially the same when the size of the modeled optic nerve was sequentially increased from 10% to 20% to 50% of the actual human optic nerve.

**Figure 7:**
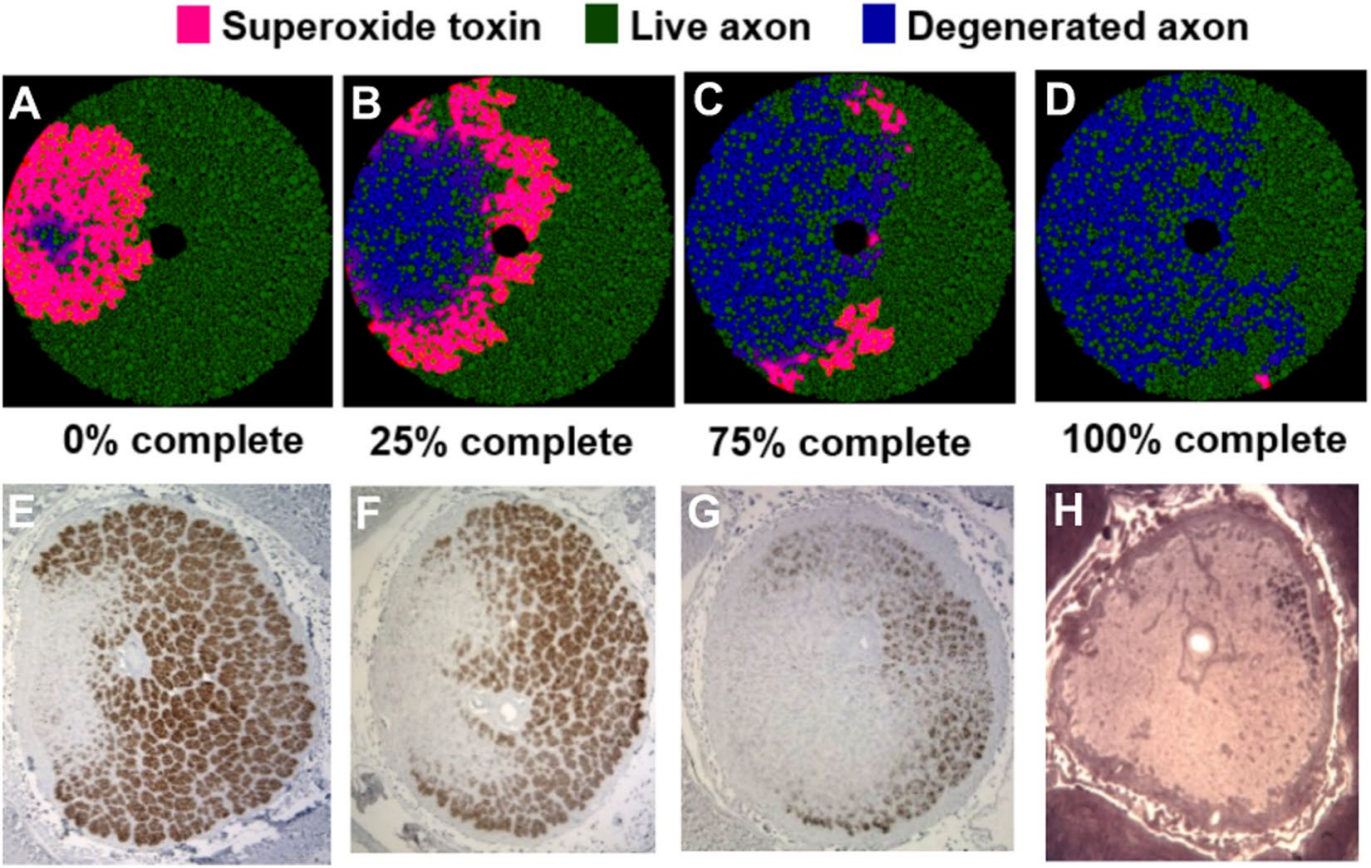
Comparison of our simulated 10% scaled optic nerve injury to *in vivo* histopathological cross-sections of actual LHON optic nerves. (**A**) Schematic of the optic nerve simulation when 25% of the simulation was completed. (**B**) Simulation at 50% completion. (**C**) Simulation at 75% completion. (**D**) Simulation at 100% completion (at steady state). (**E–H**) *In vivo* histopathological cross-sections of actual LHON optic nerves at chronologically increasing stage of the disease (photomicrographs courtesy of Dr. Alfredo Sadun).

The interaction between the anatomical axonal size distribution and the site of the initial injury provides a putative explanation for the natural history of LHON. Based on the modeled data, larger axons were more protected than smaller ones, likely due to their relatively higher scavenging activity compared to superoxide generation. The preferential effect on small axons correlates with the observation that small axons are preferentially affected in LHON, based on the substantial effects on color vision and visual acuity. Comparison of the steady state distribution of degenerated axons following an initial temporal injury with actual histopathological cross-sections of LHON optic nerves demonstrates a striking similarity (Fig. 7), and supports the preferential sparing of nasal quadrant axons. This nasal sparing was also supported by the correlation in the Garway-Heath optic nerve zone distribution between the mean deviation thresholds obtained from actual LHON visual fields (Fig. 5) and the steady state profile of simulated degenerated axons (Fig. 6). Furthermore, the nasal (zone 1) scavenging of superoxide propagation was consistent and reproducible, regardless of the location of the initial injury.

Elapsed time to steady state in the simulation reflects the time-course of spread of degeneration from initial injury to completion, the latter defined as reaching the asymptote. Different parameters resulted in different intervals to achieve steady state. Temporal injuries required longer time to reach steady state compared to nasal injuries. For example, 10 simulation runs of the 10% scaled optic nerve demonstrated a mean time to steady state of 448 ± 50 iterations and 220 ± 23 iterations for superotemporal and inferonasal injuries, respectively. This difference is explained by a greater spread of degeneration within the temporal nerve due to a larger proportion of small axons temporally and superiorly, and their greater relative sensitivity to superoxide-mediated degeneration. Assuming that in patients with acute LHON it takes one week for the degeneration to spread throughout the optic nerve with an initial temporal injury, then those patients with an initial nasal injury would have less degeneration, and it would eventually reach steady state in half that time, i.e. 3 to 4 days.

There are several limitations to this simulation. First, it was performed in two dimensions, modeling the optic nerve as a cross section, and therefore not accounting for longitudinal diffusion. Second, it did not take into account the following: (1) differences in diffusion inside glial cells and the microvasculature; (2) differences in diffusion across axonal membranes; and (3) the effects of optic nerve myelination on diffusion. Several of the chosen constants were arbitrary, because of lack of published experimental information. Finally, although there is good evidence that superoxide levels increase in LHON mutations in cybrids^15,21^ and that RGC death is affected by superoxide^17,22^, we do not know whether this is true for RGC axons, or whether a different interconvertible reactive oxygen species (e.g. hydrogen peroxide) is the axonal toxin. Future work will consist of (1) simulating the model in three dimensions, thus including longitudinal superoxide diffusion along the axons; (2) taking into account the anatomical transition from unmyelinated to myelinated axons; and (3) validating the model with data from *in vitro* and *in vivo* RGC biology.

In summary, we modeled cross-sections of LHON optic nerves, examining the effect of initial superoxide injury location on the propagation and extent of axonal degeneration. The modeled axonal size distribution was based on realistic human optic nerve histological measurements, with smaller and larger axons preferentially located temporally and nasally. The results obtained matched the clinical and pathological findings in patients with LHON, with small axon and temporal nerve degeneration being consistent with clinical observations of cecocentral scotomas and dyschromatopsia. Sensitivity analyses showed that the model was robust, realistic, repeatable, and scalable. Overall, our model and its validation with actual visual fields and pathology provides a pathophysiological mechanism for the initiation and progression of LHON^8,21^ that is consistent with the anatomical and clinical features of the disease.

## Methods

### Modeling the human optic nerve

The optic nerve was modeled as a circle superimposed on a matrix of pixels. This two-dimensional section of the human optic nerve was simulated by filling the nerve with axon bundles, where the distribution of axon sizes within each bundle varied depending on the location (superior/inferior and temporal/nasal) within the nerve. Axon cross-sections were assumed to be perfectly circular. The model resolution was set at 10 pixels per µm. The number of pixels inside a circular element was dependent on the diameter of the element itself and the fixed resolution. For example, a 1500 × 1500 matrix was used for a 10% scaled optic nerve.

Model parameters included optic nerve radius, minimum distance between two axons (i.e. clearance, reflecting space for myelin and glial cells), and distribution of axon radii. These parameters were adjusted to reflect the distribution of axons. The number of axons in the human optic nerve is 1.2 × 10^6^ ± 4.7 × 10^5^ axons^19^. In our model, a 10% scaled version of the optic nerve was mostly used, with a total of appoximately 12000 axons.

### Distribution of axon sizes

The distribution of axon radii was derived from the axon diameter histograms for normal nerves (Fig. 3 from Pan *et al*.^14^). In order to account for three different groups of axons sizes (small, medium and large), the plotted distributions were fitted with a three-component Gaussian function:

$${\sum {a}_{1}e}^{{[-\frac{x-{\mu }_{1}}{{\sigma }_{1}}]}^{2}}+{a}_{2}{e}^{{[-\frac{x-{\mu }_{2}}{{\sigma }_{2}}]}^{2}}+{a}_{3}{e}^{{[-\frac{x-{\mu }_{3}}{{\sigma }_{3}}]}^{2}}$$

The coefficients of the Gaussian fit were adjusted so that the population mean and standard deviation corresponded to that of Pan *et al*. for each of the regions of the optic nerve (Fig. 4 from^14^). The distributions for axon sizes denoted as small, medium and large axons were adjusted so that there were equal numbers of axons in each group.

### Diffusion model

The propagation of injury was simulated using an initial localized high concentration of superoxide, which then spread via passive diffusion from one axon to adjacent axons, resulting in their degeneration when the intra-axonal concentrations reached a toxic threshold. This initial injury reflects the increased levels of superoxide associated with LHON mutations in cybrid cells^15^, the likely cause of the LHON phenotype^21,23^. The concentration of superoxide within a particular element over time is a function of its concentration and that of its 4 neighboring elements, based on the following equation:

$${C}_{i,j}^{t+1}=(\begin{array}{c}{C}_{i,j}^{t}+{P}_{i,j}+\\ {D}_{i,j}^{i+1,j}({C}_{i+1,j}^{t}-{C}_{i,j}^{t})+\\ {D}_{i,j}^{i-1,j}({C}_{i-1,j}^{t}-{C}_{i,j}^{t})+\\ {D}_{i,j}^{i,j+1}({C}_{i,j+1}^{t}-{C}_{i,j}^{t})+\\ {D}_{i,j}^{i,j-1}({C}_{i,j-1}^{t}-{C}_{i,j}^{t})\end{array}){S}_{i,j}$$

where:

${C}_{i}^{t}$ = Superoxide concentration in µmol within element *i*, *j* at time *t*.

${D}_{i,j}^{m,n}$ = Unitless superoxide diffusion constant between adjacent elements *i*, *j* and *m*, *n*. The constant depends on location, i.e. whether intra-axonal or extra-axonal.

${P}_{i,j}$ = Superoxide production rate at element *i*, *j* in µmol/iteration. The activity of Na^+^, K^+^-ATPase required for axonal membrane repolarization is proportional to the amount of axonal conduction, and reflects the quantity of electrons flowing through the mitochondrial electron transport chain. With an LHON mutation, the increased superoxide production is proportional to the flow of electrons, and therefore the amount of axonal conduction. The latter is a function of the axonal surface area, which is proportional to its circumference in the slice.

${S}_{i,j}$ = Superoxide scavenging constant in µmol/iteration, assuming a first-order process and equal distribution of superoxide dismutase per unit area in the slice (equivalent to volume in the nerve). The constant varies depending on location, i.e. whether intra-axonal or extra-axonal.

The simulation steady state was considered as attained when the concentration of superoxide varied by less than 0.01 µmol over time.

Based on the histopathology from Pan *et al*.^14^, we estimated the number of surviving axons in LHON nerves to range from 35% to 40%. We used this range as a benchmark to guide our diffusion-model analyses in order to achieve realistic numbers at steady state.

### Simulation platform

The simulation was coded in C#/C++ (Microsoft Visual Studio 2016), running on an Intel Core i7-4790 CPU at 3.6 GHz. The open source CUDAfy.NET package was used to integrate NVidia CUDA with our GUI in C# [https://cudafy.codeplex.com/]. The simulation of the propagation of injury from axon to axon was massively parallel processed on a NVIDIA® GeForce® GTX 745 graphics card with 394 Cuda cores running at 793 gigaflops (billion floating operations per second).

### Analysis of simulation results

Once a steady state was reached, the data were imported into MATLAB for analysis. In order to optimize the diffusion model, a series of sensitivity analyses were performed on parameters thought likely to affect the propagation of superoxide, i.e. the superoxide intra- and extra-axonal scavenging rates and the amount of superoxide released when the axon degenerated. For each parameter, the value was varied while keeping the other parameters constant. The axonal size distribution at steady state was compared for each parameter in the sensitivity analysis.

### Simulation interface and graphical output

**Figure 8:**
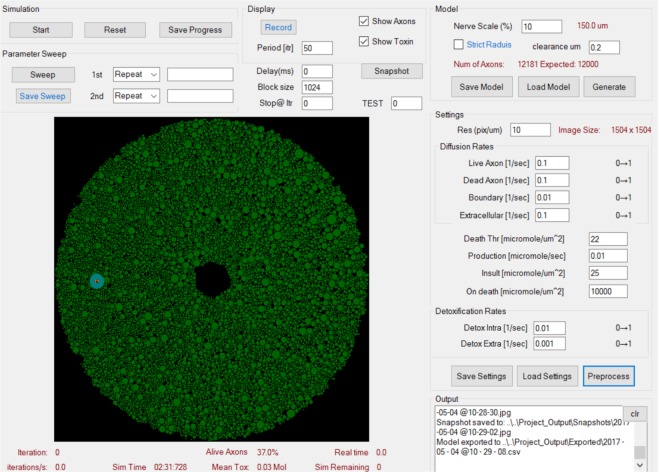
Simulation Initialization and Graphical Output. Control panel for the optic nerve modelling parameters (top right section: “Model”) and superoxide diffusion parameters (middle right section: “Diffusion and Scavenging rates”). The platform allows recording a simulation as well as taking snapshots at different time intervals. It also displays the number of iterations performed, the proportion of alive axons, total amount of superoxide in moles as well as the elapsed simulation time. A schematic of the initial state for optic nerve axons is depicted in green.

Figure 8 illustrates the interface used to control the optic nerve modelling parameters and the superoxide diffusion parameters. The platform allows recording a simulation as well as taking snapshots at different time intervals. It also displays the number of iterations performed, the proportion of live axons, the mean toxin concentration in µmol, and the simulation time in real time. Figure 1 provides a representation of the optic nerve axons in cross section at the initial state, before the simulation begins.

### Comparison of model results with data from LHON patients

Validation of the model was achieved using two types of patient-level data. First, the simulated optic nerve damage in the LHON model was mapped to the visual field that would be expected to result, using the optic nerve zone distribution from the Garway-Heath map^20^ (Supplementary Fig. 1). The zone-specific thresholds obtained from automated (Humphrey Visual Field; HVF) 30-2 SITA-FAST or FASTPAC visual fields of real LHON patients were then compared to the steady-state distribution of axons obtained from multiple simulations of a 10% optic nerve model. Second, two-dimensional images of dead vs. live axons from LHON simulations were compared to histopathological cross-sections of optic nerves from LHON patients obtained *post mortem*. Visual fields and histological sections were kindly provided by Alfredo Sadun, MD, PhD.

In order to analyze visual fields from LHON patient, each of the 76 points from the threshold sensitivity map were assigned to their corresponding Garway-Heath optic nerve zone and the mean of the thresholds for each zone was calculated. We then ran 20 different LHON optic nerve simulations using 20 different initial injury locations. For each simulation, the predicted proportion of degenerated axons in each of the Garway-Heath optic nerve zones was calculated. Finally, the amount of predicted zone abnormalities based on visual fields from LHON patients was correlated with the results of the simulations.

### Use of material from human participants

Histopathological and visual field material provided by Dr. Alfredo Sadun was obtained in accordance with relevant human subject research guidelines. Specifically, the Instutional Review Board of the University of Southern California had approved the use of these materials for research, including the post-mortem obtaining and processing of optic nerves from participants with LHON. Informed consent was obtained from all participants.

### Statistics

One-way ANOVA was used to analyze the effects of propagation surface area, speed, and proportion of degenerated axons based on size (small, medium and large). A p-value less than 0.05 was considered statistically significant.

## Supplementary information

Supplementary Data

Video 1

Video 2
