# Author Correction: Propagation and Selectivity of Axonal Loss in Leber Hereditary Optic Neuropathy

**DOI:** 10.1038/s41598-020-61398-0

**Published:** 2020-03-10

**Authors:** Razek Georges Coussa, Pooya Merat, Leonard A. Levin

**Affiliations:** 1 Department of Ophthalmology and Visual Sciences, McGill University, Montreal, Canada; 2 Department of Electrical and Computer Engineering, McGill University, Montreal, Canada; 3 Department of Neurology & Neurosurgery, McGill University, Montreal, Canada; 4 Department of Ophthalmology and Visual Sciences, University of Wisconsin, Madison, WI USA

Correction to: *Scientific Reports* 10.1038/s41598-019-43180-z, published online 30 April 2019

Supplementary files “Video 1” and “Video 2” were omitted from the original version of this Article. This has been corrected in the HTML version of the Article; the PDF version was correct at time of publication.

