# Supplementary material for: Propagation and Selectivity of Axonal Loss in Leber Hereditary Optic Neuropathy
Source: Sci Rep. 2019 Apr 30;9:6720. doi: 10.1038/s41598-019-43180-z (PMC6491426; doi:10.1038/s41598-019-43180-z)
Supplement: Supplementary file 1 — Supplementary Data [file 41598_2019_43180_MOESM1_ESM.pdf]

Propagation and Selectivity of Axonal Loss in Leber Hereditary Optic Neuropathy

Razek Georges Coussa,[1] Pooya Merat,[2] Leonard A. Levin[1,3,4,*]

[1]Department of Ophthalmology and Visual Sciences, McGill University, Montreal, Canada

[2]Department of Electrical and Computer Engineering, McGill University, Montreal, Canada

[3]Department of Neurology & Neurosurgery, McGill University, Montreal, Canada

[4]Department of Ophthalmology and Visual Sciences, University of Wisconsin, Madison, WI

Supplementary Materials

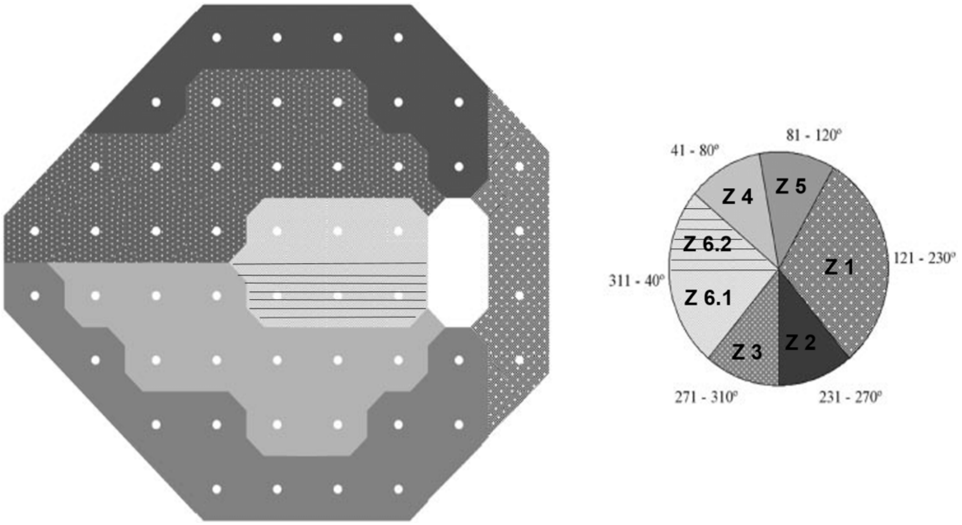

Supplementary Figure 1: Visual field (left) and optic nerve' zones (right) correspondence map. Modified from Garway-Heath DF, Poinoosawmy D, Fitzke FW, Hitchings RA. Mapping the visual field to the optic disc in normal tension glaucoma eyes. Ophthalmology. 2000;107:1809-1815.

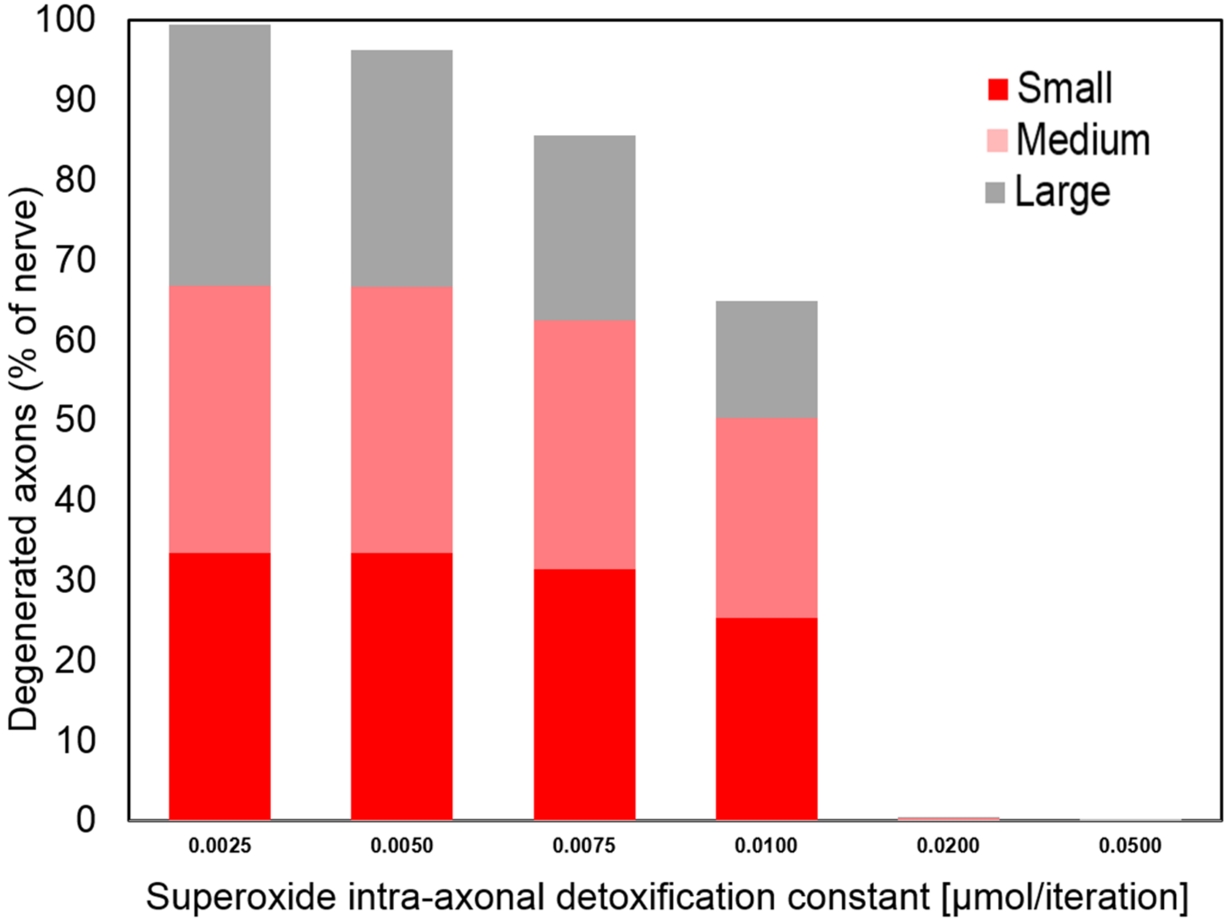

Supplementary Figure 2: Sensitivity analysis of axonal degeneration with respect to the intra-axonal superoxide scavenging constant. Data reflect axon size distribution at steady state.

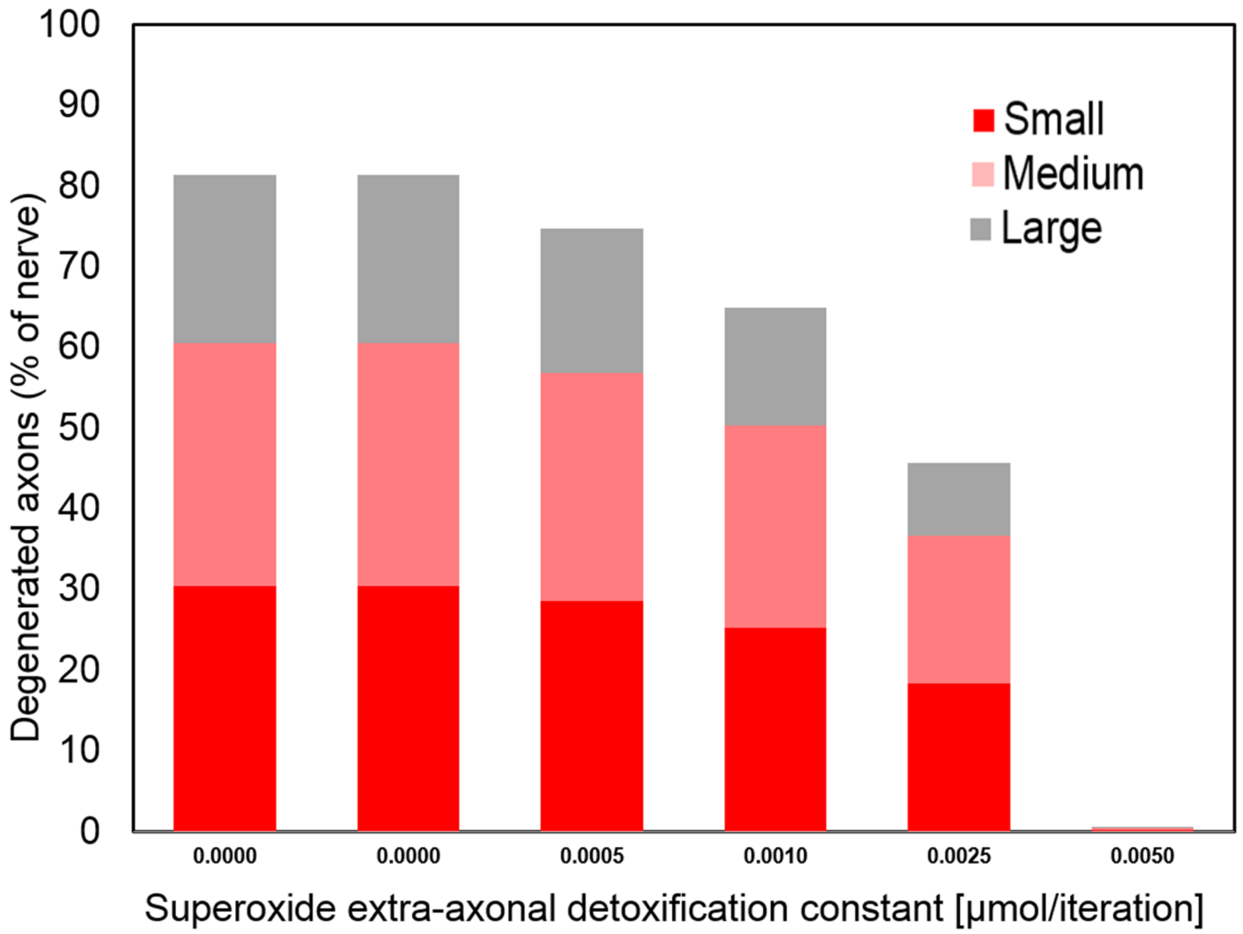

Supplementary Figure 3: Sensitivity analysis of axonal degeneration with respect to the extra-axonal superoxide scavenging constant. Data reflect axon size distribution at steady state.

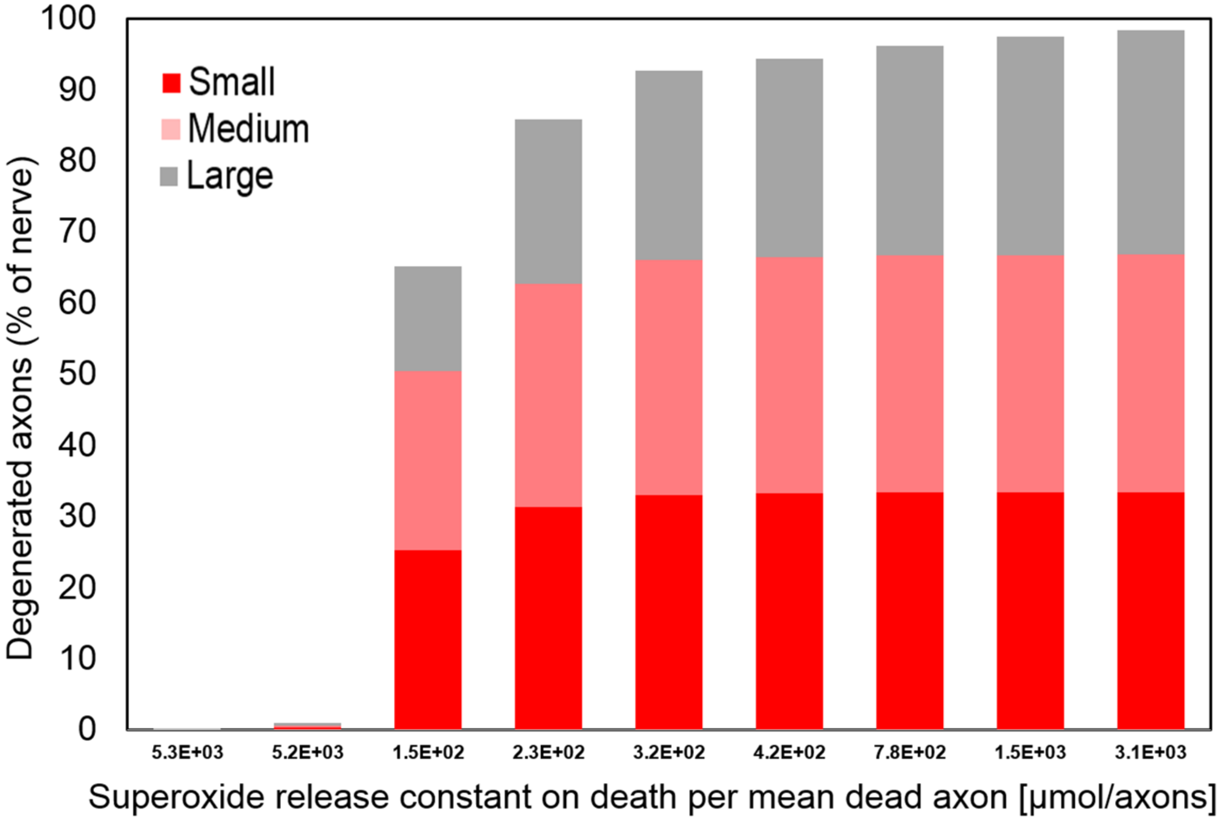

Supplementary Figure 4: Sensitivity analysis of axonal degeneration with respect to the superoxide release constant. Data reflect axon size distribution at steady state.

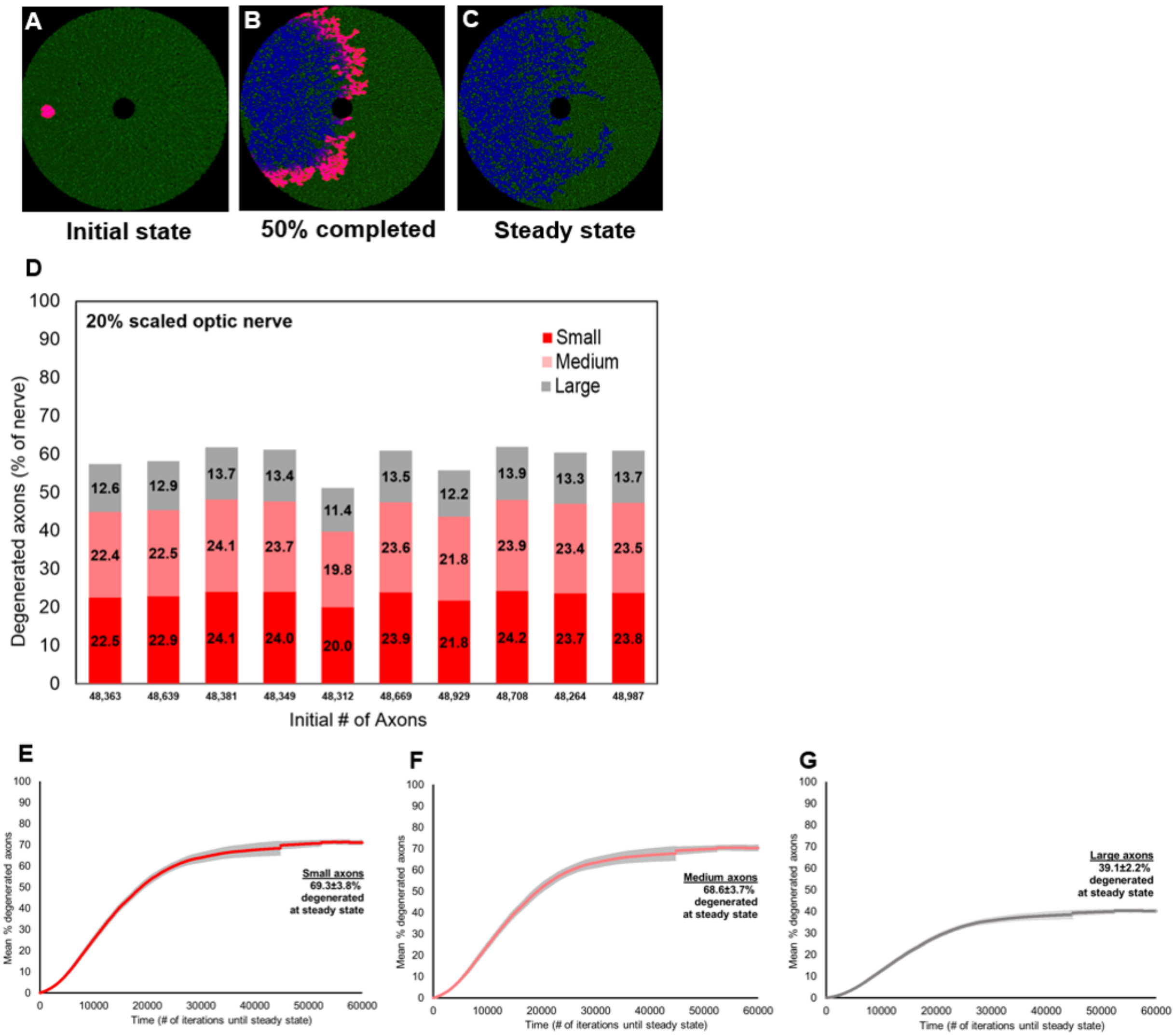

Supplementary Figure 5: Degenerated axon size distribution for 10 different 20% scaled optic nerves with constant simulation parameters and mid-temporal injury. **A.** Schematic of an optic nerve with an initial temporal injury (t = 0). **B.** Simulation 50% complete. **C.** Simulation complete (i.e. steady state). **D.** Steady-state axon size distribution for each simulation run**.** **E.** Mean proportion of small degenerated axons over time. **F.** Mean proportion of medium-sized degenerated axons over time. **G.** Mean proportion of large degenerated axons over time.

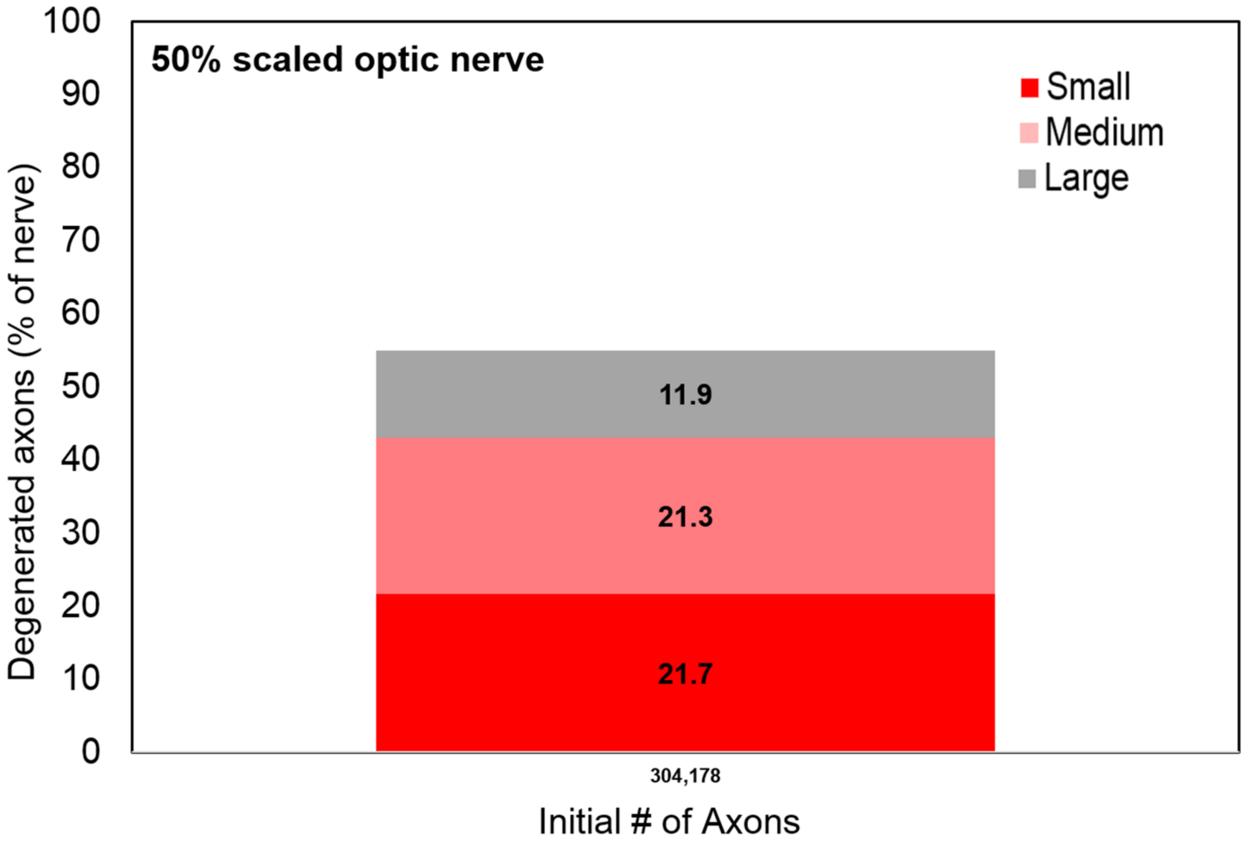

Supplementary Figure 6: Degenerated axon size distribution for a 50% scale optic nerve mid-temporal injury. Data reflect axon size distribution at steady state.

Supplementary Table 1: Constants values of the three-component gaussian function used for the initial axon size distribution

	a_n	μ_n	σ_n
**1**	0.057<br>(95%CI: 0.028-0.085)	0.730<br>(95% CI: 0.717-0.744)	0.253<br>(95% CI: 0.212-0.294)
**2**	0.0395<br>(95% CI: 0.018-0.061)	1.076<br>(95% CI: 0.888-1.265)	0.442<br>(95% CI: 0.253-0.630)
**3**	0.017<br>(95% CI: 0.012-0.022)	1.73<br>(95% CI: 1.367-2.094)	0.924<br>(95% CI: 0.708-1.141)

Supplementary Table 2: Degenerated axon size distribution as a function of varying injury location along the temporal-nasal axis.

Injury location	% Degenerated small axons	% Degenerated medium axons	% Degenerated large axons	Total % Degenerated Axons
**Most temporal**	23.99	23.95	14.08	62.02
**Temporal**	24.02	23.98	14.01	62.01
**Mid temporal**	24.01	23.94	13.98	61.93
**Mid nasal**	0.35	0.37	0.03	0.75
**Nasal**	0.24	0.32	0.23	0.80
**Most nasal**	0.16	0.06	0.05	0.27

Supplementary Table 3: Degenerated axon size distribution as a function of varying injury location along the superior-inferior axis.

Injury location	% Degenerated small axons	% Degenerated medium axons	% Degenerated large axons	Total % Degenerated Axons
**Most superior**	0.32	0.39	0.29	1.01
**Superior**	22.66	22.74	12.53	57.93
**Mid superior**	22.96	23.09	12.75	58.81
**Mid inferior**	1.42	1.35	0.95	3.72
**Inferior**	1.88	1.94	1.30	5.12
**Most inferior**	0.18	0.26	0.24	0.68

Supplementary Table 4: Degenerated axon size distribution as a function of superoxide intra-axonal scavenging.

Superoxide intra-axonal scavenging constant [μmol]	% Degenerated small axons	% Degenerated medium axons	% Degenerated large axons	Total % Degenerated Axons
0.0025	33.35	33.40	32.68	99.43
0.005	33.35	33.34	29.52	96.21
0.0075	31.32	31.13	23.10	85.54
0.01	25.28	25.03	14.59	64.89
0.02	0.11	0.16	0.04	0.30
0.05	0.02	0.07	0.02	0.11

Supplementary Table 5: Degenerated axon size distribution as a function of superoxide extra-axonal scavenging.

Superoxide extra-axonal scavenging constant [μmol]	% Degenerated small axons	% Degenerated medium axons	% Degenerated large axons	Total % Degenerated Axons
0.000001	30.48	30.13	20.83	81.44
0.00001	30.47	30.12	20.79	81.38
0.0005	28.61	28.23	17.95	74.79
0.001	25.28	25.03	14.59	64.89
0.0025	18.39	18.27	9.08	45.75
0.005	0.16	0.23	0.06	0.45

Supplementary Table 6: Degenerated axon size distribution as a function of superoxide release constant.

Superoxide release constant on degeneration [μmol]	% Degenerated small axons	% Degenerated medium axons	% Degenerated large axons	Total % Degenerated Axons
1000	0.06	0.07	0.07	0.19
5000	0.32	0.32	0.32	0.97
10000	25.31	25.16	14.76	65.23
20000	31.41	31.33	23.15	85.9
30000	33.00	33.05	26.71	92.76
40000	33.23	33.21	27.88	94.32
75000	33.38	33.38	29.41	96.17
150000	33.38	33.42	30.72	97.53
300000	33.38	33.43	31.53	98.35

Supplementary Table 7: Degenerated axon size distribution for 10 different optic nerves with constant simulation parameters (10% scaled optic nerve, mid-temporal injury).

Run Number	Initial Number of Axons	% Degenerated Small Axons	% Degenerated Middle Axons	% Degenerated Large Axons	Total % Degenerated Axons
1	12388	26.5	25.7	15.4	67.6
2	12228	24.1	23.5	14.0	61.6
3	12266	24.4	24.5	14.9	63.8
4	12195	26.2	26.2	15.7	68.1
5	11992	24.5	24.0	13.7	62.2
6	11958	23.3	22.9	13.1	59.3
7	12021	23.7	23.5	13.5	60.8
8	11833	24.8	24.4	14.1	63.3
9	12223	24.1	23.4	13.6	61.2
10	11936	22.5	22.9	13.1	58.4
**Mean**	12104 ± 169	24.4 ± 1.2	24.1 ± 1.1	14.1 ± 0.9	62.6 ± 3.0

Supplementary Table 8: Degenerated axon size distribution for 10 different optic nerves with constant simulation parameters (20% scaled optic nerve, mid-temporal injury).

Run Number	Initial Number of Axons	% Degenerated Small Axons	% Degenerated Middle Axons	% Degenerated Large Axons	Total % Degenerated Axons
1	48363	22.5	22.4	12.6	57.5
2	48639	22.9	22.5	12.9	58.2
3	48381	24.1	24.1	13.7	61.8
4	48349	24.0	23.7	13.4	61.2
5	48312	20.0	19.8	11.4	51.2
6	48669	23.9	23.6	13.5	61.0
7	48929	21.8	21.8	12.2	55.8
8	48708	24.2	23.9	13.9	62.0
9	48264	23.7	23.4	13.3	60.4
10	48987	23.8	23.5	13.7	61.0
**Mean**	48560 ± 250	23.1 ± 1.3	22.9 ± 1.3	13.1 ± 0.8	59 ± 3.3

Supplementary Table 9: Total percentage of degenerated axons per initial injury location (10% scaled optic nerve).

Initial Injury location	Proportion of Degenerated Axons
**1**	57
**2**	57
**3**	57
**4**	8
**5**	57
**6**	57
**7**	57
**8**	32
**9**	1
**10**	0.3
**11**	1
**12**	2
**13**	1
**14**	57
**15**	58
**16**	4
**17**	58
**18**	3
**19**	57
**20**	57
